# Validation of the Polish PPQ-II: sociodemographic and clinical correlates of perinatal PTSD

**DOI:** 10.3389/fpsyg.2025.1698527

**Published:** 2025-10-24

**Authors:** Anna Weronika Szablewska, Lucyna Wójcicka, Arkadiusz Prajzner, Agata Zdun-Ryżewska, Julia Burdecka, Dagmara Klasa-Mazurkiewicz

**Affiliations:** 1 Division of Obstetric and Gynaecological Nursing, Faculty of Health Sciences with the Institute of Maritime and Tropical Medicine, Medical University of Gdańsk, Gdańsk, Poland; 2 Institute of Psychology, Faculty of Pedagogy and Psychology, University of the National Education Commission, Krakow, Kraków City, Poland; 3 Division of Quality of Life Research, Department of Psychology, Faculty of Health Sciences with the Institute of Maritime and Tropical Medicine, Medical University of Gdańsk, Gdańsk, Poland; 4 Division of Gynecology and Obstetrics, Department of Gynecology, Obstetrics and Neonatology, Medical University of Gdańsk, Gdańsk, Poland

**Keywords:** women’s mental health, perinatal post-traumatic stress disorder, psychometric properties, questionnaires, cross-cultural validation

## Abstract

**Background:**

Perinatal PTSD (CB-PTSD) is a significant psychological condition that may affect mothers after childbirth, with long-term consequences for mental health. Despite growing global interest, limited research has addressed its prevalence and risk factors in Poland. This study aimed to validate the Polish version of the Perinatal PTSD Questionnaire (PPQ-II) and to explore sociodemographic and clinical correlates of CB-PTSD among Polish mothers.

**Methods:**

A cross-sectional study was conducted in December 2024 with a sample size 273 Polish mothers using the Computer-Assisted Web Interview (CAWI) method. The PPQ-II was adapted through translation, back-translation, and expert review. Psychometric evaluation included internal consistency, construct validity, and convergent validity with the PTSD-8 and DASS-21. Exploratory factor analysis (EFA) assessed the factor structure. Data analysis employed IBM SPSS 29.0 and Jamovi 2.4, with non-parametric tests examining associations with sociodemographic and clinical variables.

**Results:**

EFA revealed two main domains: Avoidance/Intrusion and Arousal explaining 62% of total variance. The PPQ-II showed good internal consistency (Cronbach’s α = 0.90) and convergent validity. Results of Polish version of the PPQ-II were observed to be positively correlated with PTSD-8 (*r* = 0.43–0.85) and DASS-21 (*r* = 0.54–0.78). Significant differences in PPQ-II scores were observed across delivery mode, birth complications, and neonatal ICU admission. Higher CB-PTSD symptoms were reported by women undergoing cesarean sections, those whose infants required intensive care, and mothers of children with congenital defects. Lower socioeconomic status was also associated with higher scores.

**Conclusion:**

The Polish adaptation of the PPQ-II demonstrates strong reliability and validity for assessing perinatal PTSD. Maternal sociodemographic and clinical factors—particularly delivery method, neonatal complications, and socioeconomic conditions—substantially influence CB-PTSD severity. These findings emphasize the need for psychosocial assessment in perinatal care and targeted support for high-risk groups to mitigate adverse psychological outcomes.

## 1 Introduction

Despite the common perception of parturition as a joyous and transformative process, research demonstrates that a significant proportion of women worldwide, ranging from 20% to 48%, report experiencing it as traumatic (Shiva et al., 2021; Benzakour et al., 2022; Dekel et al., 2023; Suarez and Yakupova, 2023). Negative perinatal experiences can lead to childbirth-related post-traumatic stress symptoms (CB-PTSS) or even full-blown childbirth-related post-traumatic stress disorder (CB-PTSD). Notwithstanding the increasing scholarly attention given to childbirth-related post-traumatic stress syndrome/disorder (CB-PTSS/PTSD), a standardized definition remains elusive. This is due in part to the fact that both definitions and diagnoses of CB-PTSS/PTSD are subject to variability depending on the criteria outlined in the current international classification of diseases. (Suarez and Yakupova, 2023; Alves et al., 2024; Nardozza et al., 2025).

The symptomatology of CB-PTSD includes reliving the traumatic birth experience (e.g., nightmares, flashbacks), persistent avoidance behaviors (e.g., avoiding birth-related thoughts or reminders), as well as heightened Arousal symptoms (e.g., irritability, difficulty concentrating) (Thiel et al., 2018; Dekel et al., 2023). Studies indicate that 4.7% of postpartum women meet the diagnostic criteria for CB-PTSD, with 12.3% experiencing significant CB-PTSS (Heyne et al., 2022; Alves et al., 2024). These numbers increase among high-risk groups, such as women who experienced traumatic deliveries or neonatal complications (Shiva et al., 2021; Suarez and Yakupova, 2023). CB-PTSS/PTSD can have severe consequences on maternal mental health, infant development, and family dynamics, even in cases where women do not meet the full diagnostic criteria for PTSD (Sandoz, 2021; Heyne et al., 2022; D’Hooghe and Kerkplein, 2023; Frankham et al., 2024). Unfortunately, CB-PTSD is not formally recognized as a distinct diagnosis in the DSM-5 (Diagnostic and Statistical Manual of Mental Disorders, Fifth Edition) or in the ICD-11 (International Classification of Diseases, 11th Revision) as a separate condition. Moreover, CB-PTSD often coexists with postpartum depression, exacerbating maternal distress (Liu et al., 2021; Silva-Fernandez et al., 2023), which can make diagnosis of the disorder much more difficult.

In the broader context of trauma-related disorders, childbirth-related post-traumatic stress disorder (CB-PTSD) can be classified within the general framework of PTSD as outlined in international diagnostic manuals. According to the International Classification of Diseases, 11th Revision (ICD-11), PTSD is characterized by three core symptom clusters: re-experiencing the traumatic event (e.g., intrusive memories, flashbacks, nightmares), avoidance of trauma-related stimuli, and a persistent sense of current threat, often manifesting as hypervigilance or exaggerated startle responses. The ICD-11 conceptualization of PTSD focuses on these core symptoms, offering a streamlined approach that distinguishes the disorder from complex PTSD (CPTSD), which involves additional disturbances in self-organization, such as emotional dysregulation and interpersonal difficulties (World Health Organization, n.d.).

In contrast, the Diagnostic and Statistical Manual of Mental Disorders, Fifth Edition (DSM-5) presents a broader PTSD framework, requiring the presence of symptoms from four clusters: Intrusion (e.g., distressing memories, flashbacks), Avoidance (e.g., avoiding reminders of the event), negative alterations in cognition and mood (e.g., persistent negative beliefs, emotional numbing), and marked alterations in Arousal and reactivity (e.g., hypervigilance, exaggerated startle response, sleep disturbances). Notably, DSM-5 also specifies that trauma exposure must involve actual or threatened death, serious injury, or sexual violence. Such exposure can occur through direct experience, witnessing the event, learning about a traumatic event affecting a close relative or friend (if violent or accidental), or repeated or extreme exposure to distressing details of trauma (e.g., emergency responders dealing with cases of severe injury or abuse) (American Psychiatric Association, n.d.).

Although neither ICD-11 nor DSM-5 recognize CB-PTSD as a distinct diagnostic entity, both frameworks provide a well-established basis for diagnosing post-traumatic stress disorder in the context of childbirth. The PTSD criteria outlined in these classifications are widely used in clinical practice to identify and assess trauma-related symptoms following childbirth. Increasing evidence suggests that many women meet PTSD criteria postpartum, particularly when childbirth is perceived as a traumatic event for themselves or their child. Thus, while CB-PTSD is not separately categorized, the existing diagnostic framework allows for the effective assessment and management of post-traumatic stress symptoms in perinatal settings.

Despite the significant impact of CB-PTSS/PTSD, it remains underdiagnosed in perinatal care settings, likely due to the lack of standardized screening measures (Alves et al., 2024). While tools for assessing PTSD exist, they primarily evaluate general PTSD symptoms rather than those specifically associated with traumatic childbirth. Evidence suggests that CB-PTSD differs in symptom presentation from other trauma-related disorders, necessitating specialized assessment instruments. For example, CB-PTSD includes childbirth-specific intrusive thoughts and nightmares, which differ from PTSD stemming from other traumatic events (Heyne et al., 2022).

Currently, Poland lacks validated tools specifically designed to assess CB-PTSS/PTSD. Existing instruments focus on general PTSD symptoms rather than capturing the nuances of birth-related trauma. This gap in perinatal mental health assessment is particularly concerning given Poland’s declining birth rate, mirroring broader European trends (Trepczyk and Szablewska, 2024). Recent research highlights the influence of biopsychosocial factors on reproductive decisions, including previous perinatal experiences, access to healthcare, and mental health concerns (Goliczewska and Szablewska, 2024). Additionally, Poland has one of the highest cesarean section rates in Europe (Szablewska et al., 2023), a factor associated with increased CB-PTSS/PTSD risk (Hüner et al., 2024). While Poland’s Perinatal Care Standards aim to ensure high-quality maternal healthcare (Minister Zdrowia, 2018), gaps remain in addressing the psychological impact of childbirth trauma (Fundacja Rodzić po Ludzku., 2021; Ministerstwo Zdrowia, 2024). Given the aforementioned phenomenon, a systemic deficiency within Polish perinatal care is readily apparent.

The PPQ-II has demonstrated strong psychometric properties across multiple cultural contexts (Alves et al., 2024; Nardozza et al., 2025). However, no validated Polish version of the PPQ-II currently exists.

The objective of our study is to assess the reliability and validity of an adapted version of the Perinatal PTSD Questionnaire (PPQ-II) and to address a critical gap by examining the association between selected sociodemographic and clinical predictors and the manifestation of PP-PTSD symptoms. As researchers, we posit that the findings will be instrumental in developing targeted screening strategies and designing effective interventions, thereby supporting comprehensive maternal mental health care and positively influencing both maternal and child health outcomes.

## 2 Materials and methods

### 2.1 Study design and participants

This cross-sectional observational study evaluated the factor structure and psychometric properties of the Polish version of the Perinatal PTSD Questionnaire (PPQ-II) and examined sociodemographic and clinical correlates of childbirth-related PTSD (CB-PTSD). The study was conducted in accordance with the Declaration of Helsinki and approved by the Bioethics Committee of the Medical University of Gdańsk (KB/540/2024).

A total of 273 mothers participated between 12 and 31 December 2024, recruited via online platforms and social media groups for mothers (approx. 28,000 members). Inclusion criteria were maternal age > 18 years, Polish language proficiency, and child aged 3–18 months. Exclusion criteria included postpartum mood disturbances (< 2 months postpartum), severe psychiatric or cognitive disorders, and serious chronic illnesses potentially affecting participation. The input data, including self-reported prior psychiatric diagnoses, were reviewed by clinical experts to ensure correct application of the exclusion criteria. The digital informed consent procedure involved presenting participants with a detailed information sheet outlining the study’s purpose, procedures, risks, benefits, and data handling, followed by an active affirmation of consent via a dedicated checkbox before accessing the survey. Participants could not proceed to the survey without providing explicit consent.

### 2.2 Data collection

#### 2.2.1 Sociodemographic, medical, and obstetric questionnaire

A custom-designed self-report questionnaire collected information on sociodemographic characteristics (e.g., age, education, income), obstetric history (e.g., pregnancy complications, mode of delivery), and medical background (e.g., mental health history, previous trauma exposure). The online form incorporated specific expert-designed questions to ensure broad accessibility and minimize the exclusion of vulnerable groups, thereby optimizing participant inclusion based on study criteria.

#### 2.2.2 Postpartum PTSD Questionnaire II (PPQ-II)

Childbirth-related post-traumatic stress disorder symptoms were measured using the Postpartum PTSD Questionnaire II (PPQ-II). This 14-item instrument assesses three symptom clusters:

- Intrusiveness (e.g., nightmares, flashbacks)
- Avoidance behaviors (e.g., avoidance of reminders, emotional numbing)
- Hyperarousal (e.g., irritability, difficulty concentrating).

Its structure initially refers to the DSM-IV criteria (American Psychiatric Association, 1994). Unlike the original version created by DeMier et al. (1996) in the revised version by Callahan et al. (2006) each item was rated on a five-point Likert scale ranging from 0 (“never”) to 4 (“frequently and for more than a month”). Higher total scores indicate greater PTSD symptom severity. Permission to use and validate the PPQ-II in Poland was obtained from the original authors (Callahan et al., 2006). The Polish version of the PPQ II questionnaire has been included in Supplementary Appendix 1.

#### 2.2.3 PTSD-8

The PTSD-8 is a validated Polish-language screening tool for post-traumatic stress disorder, consisting of eight items corresponding to DSM-5 PTSD criteria. Responses were collected using a four-point Likert scale. Permission to use the Polish adaptation of this measure was granted by the original adaptation team (Mazur et al., 2024).

#### 2.2.4 Depression, anxiety, and stress scales (DASS-21)

Symptoms of depression, anxiety, and stress were assessed using the DASS-21, a widely used self-reporting scale available in the public domain. The 21-item version includes three subscales, each with seven items, rated on a four-point Likert scale from 0 (“did not apply to me at all”) to 3 (“applied to me very much, or most of the time”). Higher scores indicate greater symptom severity.

#### 2.2.5 Adaptation and validation process

The adaptation process of the PPQ-II followed international guidelines for cross-cultural validation. First, the original version was translated into Polish by two independent bilingual psychologists. A reconciled version was created and then reverse-translated into English by a separate bilingual expert to ensure semantic consistency with the original. A panel of midwifery and psychology experts reviewed the translated items to confirm conceptual accuracy and cultural appropriateness.

The psychometric evaluation included an assessment of the adaptation for internal consistency, construct validity, and convergent validity with PTSD-8 and DASS-21.

#### 2.2.6 Re-test

In order to confirm the test’s stability over time, a retest procedure was conducted. Mothers were invited to participate again in the study to ensure consistency and facilitate the merging of data. A total of 33 responses were received from the 90 participants who had initially agreed to be contacted for a follow-up. This process allowed for the validation of the instrument’s reliability and the assessment of the temporal stability of the results.

### 2.3 Statistical analysis

The analyses were conducted using IBM SPSS 29.0 and Statistica 13.3 software. The overall statistical analysis was set at an *Alpha* level of .05. The data analysis chapter was subdivided into sections assessing relevance, reliability, standardization of results, and evaluating the relationship between the tool results and selected demographic and medical factors.

Following the authors of the Spanish (Hernández-Martínez et al., 2021), Portuguese (Alves et al., 2024), English (Quinnell and Hynan, 1999), and other language versions [e.g., (Park et al., 2016; Liu et al., 2021)] an exploratory factor analysis was used in the assessment of relevance. In evaluating the Polish version, the lack of consistency in the number of factors and item breakdown prevented the use of a confirmatory method, in favor of an exploratory approach. Promax rotation was used when extracting the components, which needed to be correlated. The number of factors was assessed using the Cattell and Kaiser criteria. The factor analysis was preceded by testing the assumptions of multivariate normality (the lack of which led to the choice of the principal axis method), the appropriate number of observations using the KMO test, and the sphericity of the data with the Bartlett test. The reliability of the measures was assessed using Cronbach’s alpha (α*C*) and MacDonald’s omega (ω*M*) coefficients, with the values of these coefficients presented after the removal of the PPQ items. An additional aspect of the tool’s stability assessment was the Pearson correlation coefficient of the PPQ-II scores with the 3-month retest. The results obtained were standardized using a standard 10-point scale.

In the domain of analysis with maternal and child sociodemographic and clinical factors, non-parametric Mann-Whitney *U* and Kruskal-Wallis *H*-tests were employed, supported by Glass’ biserial correlation (*r*_*g*_), and the epsilon-squared (ε^2^), effect sizes coefficients, respectively. Assessment of correlation in this domain necessitated using Spearman’s rank coefficient (*r*_*s*_). Non-parametric tests were selected because of the lack of equality of the groups under comparison, ordinal scales of measurement (e.g., number of pregnancies and number of births), and the non-normality of the distributions.

## 3 Results

### 3.1 Relevance

**FIGURE 1:**
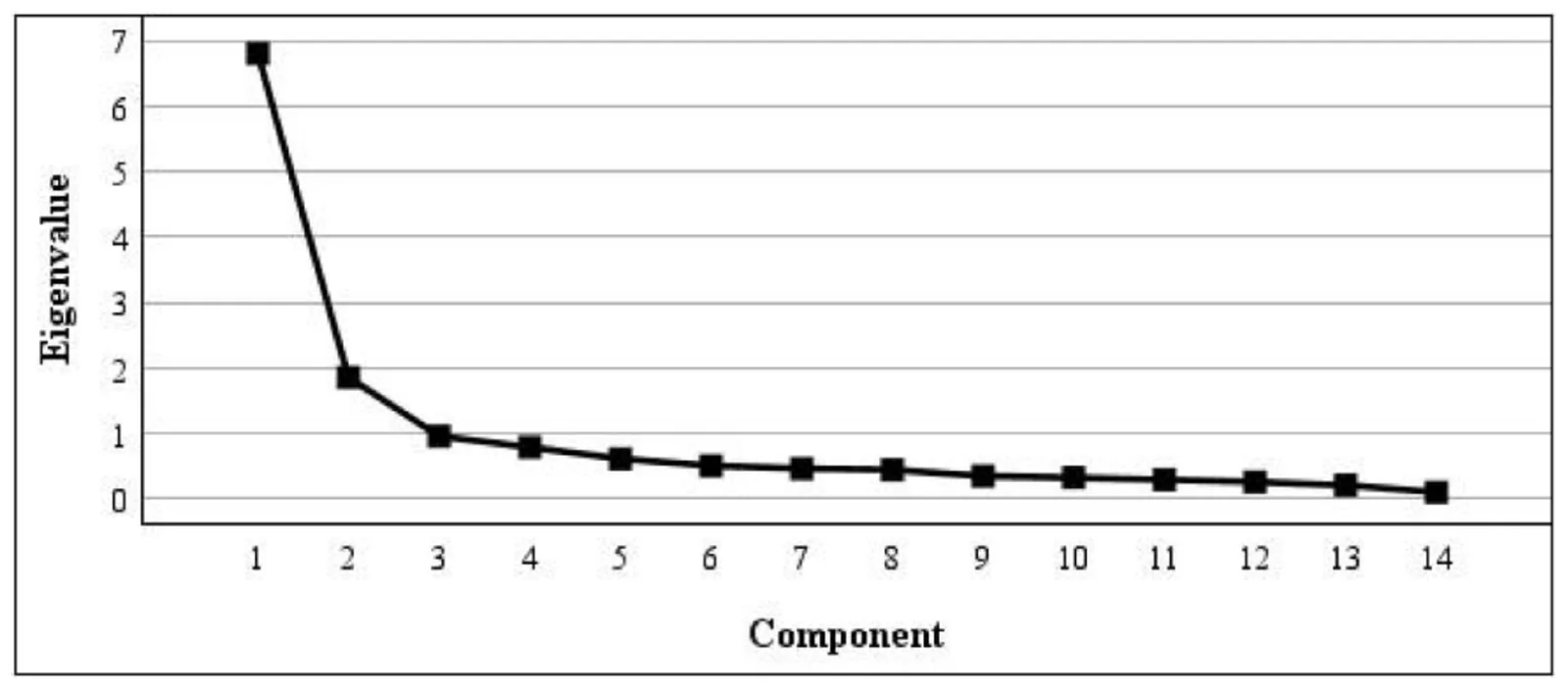
Component deposition of the Polish Perinatal PTSD Questionnaire (PPQ-II) measurement. Principal axis factoring with promax rotation was used.

In order to assess the theoretical relevance of the Polish PPQ-II version we used exploratory factor analysis (EFA) with the principal axis method. A non-orthogonal promax rotation was used in the model, which allowed the extraction of factors with a high degree of intercorrelation. The high KMO test value (0.90) indicated that the sample (*N* = 273) was sufficiently large (Field, 2018). Bartlett’s test of sphericity results also confirmed the adequacy of the correlation matrix proposed in the model, χ2(91) = 2394.94, *p* < 0.001. The scatter plot (Figure 1) shows two primary scales.

Cattell’s factor extraction criterion (Figure 1) and Kaiser’s criterion (Table 1) were equally met (Field, 2018). The results of the factor analysis indicated that two factors could be extracted. Table 1 shows that Factors 1 and 2 explained 49% and 13% of the variance, respectively. Together, the two factors explained 62% of the variance in performance.

**TABLE 1:** Explained variance of the Polish Perinatal PTSD Questionnaire (PPQ-II) measurement components.

Component	Eigenvalue	Explained variation percentage (single)	Explained variation percentage (cumulated)
1	6.80	48.58	48.58
2	1.86	13.26	61.85
3	0.96	6.85	68.70
4	0.79	5.65	74.35
5	0.62	4.40	78.74
6	0.51	3.63	82.37
7	0.47	3.33	85.70
8	0.45	3.19	88.89
9	0.35	2.51	91.40
10	0.33	2.34	93.74
11	0.30	2.12	95.86
12	0.26	1.87	97.73
13	0.21	1.51	99.24
14	0.11	0.76	100.00

Principal axis factoring with promax rotation was used.

A promax rotation was employed to evaluate the factor loadings of the PPQ-II items, with a summary of the results provided in Supplementary Appendix 2. The Avoidance and Intrusion scale demonstrated the highest loadings on items 1–5 and 14, whereas the Arousal domain was primarily represented by items 6–13. Additionally, Supplementary Appendix 2 presents the content of the questions in their original language alongside their Polish translations.

Derived from the factor loadings, both the Arousal domain and the Avoidance and Intrusion domain scores demonstrated a strong correlation (*r* = 0.58, *p* < 0.001). Furthermore, the PPQ-II total score exhibited very strong correlations with the Arousal (*r* = 0.93, *p* < 0.001) and Avoidance and Intrusion (*r* = 0.84, *p* < 0.001) domains. Criterion relevance was assessed using the PTSD-8 and DASS-21 tools. The relationship between the Polish PPQ-II results and established measures of post-traumatic stress, depression, anxiety, and stress is summarized in Table 2.

**TABLE 2:** Correlation analysis of Polish Perinatal PTSD Questionnaire (PPQ-II) with Short PTSD Inventory and Depression, Anxiety and Stress Scale results.

Tested variables		PPQ-II					
		Arousal		Avoidance and intrusion		Total score	
		*r*	*P*	*r*	*P*	*r*	*P*
Short PTSD Inventory (PTSD-8)	Intrusion	0.49	< 001	0.81	< 0.001	0.69	<0.001
	Avoidance	0.43	< 0.001	0.76	< 0.001	0.63	< 0.001
	Hypervigilance	0.58	< 0.001	0.64	< 0.001	0.68	< 0.001
	Total score	0.57	< 0.001	0.85	< 0.001	0.77	< 0.001
Depression, Anxiety and Stress Scale (DASS-21)	Depression	0.76	< 0.001	0.54	< 0.001	0.75	< 0.001
	Anxiety	0.66	< 0.001	0.62	< 0.001	0.72	< 0.001
	Stress	0.78	< 0.001	0.54	< 0.001	0.76	< 0.001

*R*, Pearson’s coefficient; *p*, significance.

The total scores of the Polish versions of the PPQ-II questionnaire exhibited strong associations with post-traumatic stress disorder scales. The Avoidance and Intrusion domain demonstrated a very strong correlation with PTSD scores, whereas the Arousal domain displayed a moderate to strong relationship with PTSD. Additionally, depression, anxiety, and stress scores showed strong to very strong correlations with the total PPQ-II scores.

### 3.2 Reliability

A consistency analysis of the Polish version of the PPQ-II in the areas of Arousal (αC = 0.86, ωM = 0.87), Avoidance and Intrusion (αC = 0.91, ωM = 0.91), and the total score (αC = 0.92, ωM = 0.92) indicated very high reliability. As shown in Supplementary Appendix 2, removing specific items from the PPQ-II did not improve reliability values. The exceptionally high reliability of the Polish version aligns with the similarly high values (αC = 0.90, ωM = 0.88, or αC = 0.90) reported for the Spanish (Hernández-Martínez et al., 2021), Portuguese (Alves et al., 2024), and English-language translations (Quinnell and Hynan, 1999).

The reliability values obtained, along with a detailed reliability assessment after removing PPQ-II questions, provide compelling evidence that the prepared version measures accurately. Reliability of the Polish PPQ-II results was further examined through a correlation analysis with the 3-month follow-up measure, as presented in Table 3.

**TABLE 3:** Correlation analysis of Polish Perinatal PTSD Questionnaire (PPQ-II) results with three months follow-up measurement.

PPQ-II	PPQ-II follow-up	
	*r*	*P*
Arousal	0.82	< 0.001
Avoidance and intrusion	0.91	< 0.001
Total score	0.90	< 0.001

Number of follow up observations *N* = 34. *r*, Pearson’s coefficient; *p*, significance.

Despite implementing follow-up calls to participants in small groups, a very high correlation was observed among the PPQ-II measures. The Polish version of the PPQ-II can be regarded as a stable measure.

### 3.3 Standardization

Despite the limited sample size, norms were developed for the Polish version of the PPQ-II. In light of the issue with the normal distribution of the total score [K-S(273) = 0.11, *p* < 0.001], the development of normative values was preceded by a non-linear transformation of the scores (Brzeziński, 2023). The transformed results, incorporating the continuity correction, were then converted to a standardized scale (Supplementary Appendix 2).

The analysis demonstrated that individuals who scored low (s-10 scores 1–3), moderate (s-10 scores 4–7), and high (s-10 scores 8–10) overall scores differed significantly on the Polish version of the PPQ-II [H(2) = 187.80, *p* < 0.001, ε^2^ = 0.69]. A *post hoc* analysis using Tukey’s test for unequal groups, shown in Table 4, confirms the significance of the differences between groups.

**TABLE 4:** *Post hoc* analysis of the Polish Perinatal PTSD Questionnaire (PPQ-II) total scores, broken down into groups distinguished in the PPQ-II standardization process.

Interpretation group	*N*	*M*	SD	Tukey’s test significance		
				1	2	3
Low (1)	42	3.31	1.91	–	–	–
Moderate (2)	183	17.16	7.50	< 0.001	–	–
High (3)	48	40.71	6.49	< 0.001	< 0.001	–

*N*, number of observations; *M*, mean; SD, standard deviation.

The limited number of observations in the sample necessitates the provisional nature of the proposed norms. However, it is noteworthy that the proposed breakdown and interpretation of PPQ-II scores effectively differentiates between the severity of perinatal post-traumatic stress disorder.

### 3.4 Perinatal post-traumatic factors

#### 3.4.1 Sociodemographic factors

A secondary yet pivotal component of the evaluation of the Polish version of the PPQ-II questionnaire pertains to its connections with specific sociodemographic and clinical factors concerning mothers and their children. The initial set of variables analyzed encompassed sociodemographic characteristics, as illustrated in the Tables in Supplementary Appendix 3. The mother’s educational level did not exert a significant influence on Polish PPQ-II scores. Additionally, no significant differences in PPQ-II scores were observed between women in informal relationships and those in married relationships. The study’s findings suggest that women with moderate and low economic status exhibited higher PPQ-II scale scores, while those with high social status demonstrated lower overall PPQ-II scale and component scores. The analysis revealed no significant differences in PPQ-II scores between women residing in rural and urban areas (Supplementary Appendix 3). An examination of the relationship between the number of pregnancies or children born and the total score and subscores of the Polish version of the PPQ-II reveals a low or very low correlation (Supplementary Appendix 3). Only the Avoidance and Introversion domain significantly correlated with the number of children born, although the relationship was low. The mother’s age was found to have no significant relationship with PPQ-II values. Similarly, the economic situation did not permit drawing definitive conclusions regarding the significance of differences, as indicated in Supplementary Appendix 3.

#### 3.4.2 Clinical mother-related factors

The study also examined the relationship between selected maternal health factors and the results of the Polish version of the PPQ-II. The findings indicate that the effect of the presence of a complication (curettage) was low to moderate. It was observed that women who underwent curettage during labor attained significantly higher PPQ-II scores (Supplementary Appendix 4). Furthermore, the assessment of hospital amenities was found to be a significant factor, with the effects rated as moderate. Conversely, the absence of hospital amenities was associated with significantly higher PPQ-II scores.

Furthermore, the pregnancy termination method was a significant factor. As demonstrated in Supplementary Appendix 4, women who underwent surgical delivery exhibited considerably higher PPQ-II scores. A comprehensive evaluation of this domain was also conducted, differentiating between surgical delivery types (Supplementary Appendix 4).

In addition, the analysis revealed that the mode of pregnancy completion played a pivotal role in differentiating PPQ-II values across different types of operative delivery. Notably, cesarean section in the first stage resulted in significantly higher scores compared to natural labor for all PPQ-II scores. Furthermore, in the Avoidance and Intrusion domain, a cesarean section in the second stage led to higher scores compared to natural labor. It is important to note that women with natural childbirth and those who had elective cesarean section scored similar PPQ-II values.

In the area of maternal health, additional factors were also analyzed. The analysis revealed that intrapartum complications, such as the Kristelle’s maneuver or the shoulder release maneuver, the occurrence of near-term complications (hemorrhage), further-term complications including extensive perineal trauma, wound healing issues, post-cesarean section complications, or ultimately prolonged hospital stays, were found to be poor correlates of PPQ-II scores. A comprehensive summary of the indicated analyses is presented in full in Supplementary Appendix 4. In addition, the occurrence of a previous miscarriage also showed low effects for PPQ-II areas. As outlined in S4Appendix, the analyses pertaining to the remaining maternal health factors proved to be largely non-statistically significant. This may be attributable to the limited number of observations available within the respective groups. These domains necessitate further investigation, incorporating a more substantial dataset.

#### 3.4.3 Clinical child-related factors

The final point of the data analysis was the comparison of PPQ-II values with selected child health factors. As demonstrated in Supplementary Appendix 4, women giving birth to babies at term were characterized by significantly lower PPQ-II scores. The effects of a child’s congenital defect on the PPQ-II scores were moderate. Significantly higher PPQ-II scores were achieved by mothers of children with congenital malformations.

The necessity for neonatal patients to be hospitalized in an intensive care unit emerged as a salient differentiating factor in the analysis of PPQ-II values. The impact of this factor was found to be modest in the Arousal domain but pronounced in the Avoidance and Intrusion domain. A statistically significant correlation was observed between the admission of newborns to an intensive care unit and higher PPQ-II scores among the study’s female participants. The necessity for additional medical care is further delineated in Supplementary Appendix 4, which provides a comprehensive overview of the demand for medical, short-term, and long-term care.

Regarding intensive care hospitalization, the factors associated with the need for additional medical care following discharge were more pronounced in the Avoidance and Intrusion domain compared to the Arousal domain. However, the necessity for additional care outside the hospital emerged as a significant determinant across all PPQ-II scores. Women whose children required long-term medical care post-delivery exhibited the highest PPQ-II scores across all scales. In contrast, no significant differences were observed between women whose children did not need additional medical care and those whose children required short-term care.

## 4 Discussion

The primary aim of this study was to assess the factor structure and psychometric properties of the PPQ-II with a sample of Polish mothers. A further significant objective of this investigation was to evaluate the sociodemographic and clinical determinants associated with the manifestation of perinatal PTSD symptomatology. These factors were categorized into two distinct domains: maternal variables and child-related determinants. Integrating screening tools like the PPQ-II into routine obstetric and gynecological care, alongside an evaluation of sociodemographic and clinical risk and protective factors, could support early identification of at-risk individuals. Such an approach may help reduce the risk of traumatic childbirth experiences and promote better maternal psychological well-being. Building on this rationale, it is important to note that the demand for further research and validated screen-ing tools is not limited to Poland. Instead, it reflects a broader trend observed across Central and Eastern Europe, where childbirth-related trauma has increasingly become a focus of maternal mental health re-search. For instance, the Lithuanian validation of the City Birth Trauma Scale confirmed the relevance of standardized PTSD screening among postpartum women (Riklikienë et al., 2025). Slovak data from the INTERSECT project have identified overlapping risk factors for postpartum depression and PTSD, empha-sizing the importance of integrated screening approaches (Ďuríčeková et al., 2024). Furthermore, a recent multicenter study involving Poland, Slovakia, and Lithuania found clinical CB-PTSD rates ranging from almost 2%–6%, again pointing to the urgent need for culturally adapted screening tools (Murawska et al., 2025). In light of these regional findings, our own validation study adds to this growing evidence base by demon-strating that the PPQ-II is both reliable and valid in the Polish context. Aligning with prior validation studies, the results reaffirm the reliability and validity of the PPQ-II, while also highlighting several important considerations discussed further in the paper.

### 4.1 The polish validation of the PPQ-II

The adaptation of the Perinatal PTSD Questionnaire II (PPQ-II) into the Polish language was conducted in accordance with internationally recognized guidelines for cross-cultural validation. The translation, reverse-translation, and expert evaluation process ensured semantic consistency with the original version while maintaining cultural appropriateness for the Polish population (Gjersing et al., 2010; Ozolins et al., 2020).

To assess the psychometric properties of the Polish PPQ-II, we estimated its basic parameters, including reliability and validity. The Polish adaptation demonstrated excellent psychometric characteristics, with high internal consistency for the total score, as well as strong reliability for its subscales. These values are comparable to the previously validated Spanish (Hernández-Martínez et al., 2021), Portuguese (Alves et al., 2024), and English (Quinnell and Hynan, 1999) versions.

The analysis of criterion and convergent validity demonstrated strong correlations between PPQ-II total scores and established measures of post-traumatic stress disorder, depression, anxiety, and stress, confirming that the instrument effectively captures the severity of perinatal PTSD symptoms. Similar relationships have been confirmed by multiple authors, highlighting significant associations between PTSD symptoms and experiences of stress, anxiety, and depression (Gjersing et al., 2010; Ozolins et al., 2020; Khsim et al., 2022). Furthermore, test-retest reliability demonstrated excellent temporal stability, with a highly significant correlation over a 3-month interval.

In addition, we conducted a normative classification to establish preliminary cutoff values for PTSD severity. Despite sample size limitations, these findings allowed for the classification of PTSD severity levels, with significant differences observed between low, moderate, and high PTSD groups. However, these standardization attempts should be interpreted with caution, as the achieved norms remain provisional and require further validation to ensure their robustness and applicability across more diverse clinical and sociodemographic groups.

Overall, these findings suggest that the Polish adaptation of the PPQ-II is a reliable and valid instrument for assessing perinatal PTSD in Polish mothers. The tool enables accurate identification of individuals at risk and reinforces the necessity of psychological screening in perinatal care. Given the lack of validated instruments specifically designed to assess PTSD related to childbirth trauma in Poland, our study provides a methodologically verified tool with robust psychometric properties. The Polish PPQ-II can serve as a reliable screening instrument for both scientific research and clinical practice, facilitating the early identification of individuals at risk for perinatal PTSD. Its application in perinatal care settings could contribute to improved psychological support and targeted interventions for affected mothers. However, further research is warranted, particularly in the area of normative validation, to refine the interpretative framework and ensure its applicability across diverse clinical and sociodemographic groups.

Finally, we examined the factorial structure of the questionnaire to verify its construct validity. The high Kaiser-Meyer-Olkin (KMO) test value (0.90) and the statistically significant Bartlett’s test of sphericity confirmed the suitability of the dataset for exploratory factor analysis (EFA). Our findings indicate that, unlike the study by Callahan et al. (2006), the PPQ-II contains of two rather than three factors, which is consistent with results from the Italian (Nardozza et al., 2025), Portuguese (Alves et al., 2024), Turkish (Akik and Batigun, 2020), and French (Pierrehumbert et al., 2004) versions of the scale. The model proposed in our own study attributed 62% of the performance variance to the interaction of both factors (Table 1), which is more than in the previously cited adaptations with two-factor models. Additionally, unlike many previous adaptations, in our study none of the original 14-item construct of the tool was removed, while maintaining a high consistency of structure (Supplementary Appendix 2).

Variability in the factor structure of the PPQ-II has been observed in previous research, both in terms of the number of factors and the distribution of items. While some validation studies support the original three-factor model (Hernández-Martínez et al., 2021; Park et al., 2016) the assignment of items to specific factors differs across studies. Even in the original research, unexpected item loadings were reported (e.g., item 2, related to distressing memories, loaded onto the Avoidance factor (Callahan et al., 2006). Therefore, a key difference compared to the original version is the integration of the “Intrusion” factor into the “Avoidance” factor. Which confirms previous scientific reports that women who have intrusive memories of childbirth often resort to avoidance behaviors as a coping mechanism to cope with trauma-related stimuli (Dekel et al., 2017). These structural inconsistencies may stem from methodological differences across studies and suggest that the original three-factor model may not be universally applicable. Further studies are recommended.

Although the two-factor structure of the tool is consistent with other cross-linguistic validation of the same tool with two-factor structures, it has a theoretical limitation as it does not fully reflect the grouping of PTSD diagnostic criteria in DSM-IV, which was the basis for the original version of the PPQ-II. However, our findings confirm that the factor structure of the PPQ-II makes it relevant for detecting general PTSD symptoms according to the updated DSM-5 criteria. Additionally, considering the ICD-11 diagnostic criteria for PTSD, which emphasize a broader understanding of trauma-related symptoms, the PPQ-II remains a valuable screening tool for identifying global PTSD symptoms. Given that the PPQ-II is intended as a screening tool for the detection of general PTSD symptoms, it is recommended to use the total score in clinical practice rather than relying on individual subscales.

### 4.2 Sociodemographic and clinical factors of perinatal trauma in Poland

The second aim of our study was to identify the risk factors associated with the onset of CB-PTSD symptoms throughout the perinatal period. This is one of the key strengths of our research, as few studies simultaneously explore the impact of sociodemographic and clinical factors, both maternal and child-related, on the development of maternal CB-PTSD.

Interestingly, we found that maternal educational level and relationship status did not significantly influence PPQ-II scores, suggesting that these variables may not be as critical in predicting CB-PTSD symptoms in our sample. This contrasts with some previous research, which has highlighted the potential impact of lower education levels and informal relationship statuses on trauma-related mental health outcomes (Nardozza et al., 2025).

We observed that women with lower economic status scored higher on the PPQ-II, while those with higher social status showed lower scores. This finding is consistent with existing literature that links lower socio-economic status with greater vulnerability to mental health issues, including PTSD, following childbirth (Ahsan et al., 2023). However, our analysis revealed no significant differences between rural and urban mothers, suggesting that geographic location may not play a crucial role in determining the risk of CB-PTSD in the Polish context.

Additionally, the number of children and the number of pregnancies showed very low correlations with PPQ-II scores, indicating that these variables may not significantly influence the development of P-PTSD symptoms. However, we did find that the Avoidance and Introversion areas of the PPQ-II correlated with the number of children, although this relationship was weak. This finding suggests that factors related to maternal experience and coping mechanisms may have a more substantial impact on symptomatology than factors such as the number of previous pregnancies.

We also found no significant relationship between maternal age and PPQ-II scores, which diverges from findings in some other studies that suggest younger mothers are at higher risk for developing PTSD symptoms following childbirth (Ahsan et al., 2023; Yakupova et al., 2023). This lack of association could reflect differences in sample characteristics or may suggest that other factors, such as the availability of support networks and emotional resources, could play a more significant role in shaping maternal mental health outcomes.

Finally, while our study did not find significant effects of economic status or history of previous miscarriages on PPQ-II scores, these factors should be considered in future research with larger and more diverse samples. The lack of significant findings may be due to the relatively small size of certain subgroups within our sample, such as women from economically disadvantaged backgrounds or those with a history of previous abortions. Further investigation into these factors may help refine our understanding of the specific risks and protective factors for maternal CB-PTSD.

In our study, we explored the impact of various maternal health factors on the development of P-PTSD symptoms, as measured by the Polish version of the PPQ-II. A significant finding was the association between complications during delivery, such as curettage, and higher PPQ-II scores. Women who underwent curettage exhibited notably higher scores in both the total scale and the subscales of Arousal, Avoidance, and Intrusion. This suggests that more invasive or traumatic interventions during childbirth may increase the risk of developing symptoms of post-traumatic stress. These results are in line with previous studies which have found that surgical interventions, particularly those considered to be more physically invasive, can have a profound psychological impact on mothers, potentially leading to higher levels of PTSD symptoms following childbirth (Cirino and Knapp, 2019; Khsim et al., 2022; Horsch et al., 2024) and lower satisfaction from childbirth (Riklikienë et al., 2025). Furthermore, the assessment of hospital amenities during delivery revealed that the lack of adequate facilities was associated with significantly higher PPQ-II scores. This highlights the importance of the physical environment during childbirth in mitigating stress and trauma. Although there are studies focusing on protective factors for PTSD, most of them emphasize the quality of care and support provided by the healthcare staff rather than examining the impact of hospital amenities themselves (Cirino and Knapp, 2019; Khsim et al., 2022). The role of hospital facilities and their influence on maternal mental health requires further exploration, as there is a lack of research specifically addressing this aspect. Hospital settings that lack proper amenities, including emotional support and physical comfort, might exacerbate feelings of helplessness and vulnerability, further increasing the risk of developing PTSD symptoms. Regarding the mode of delivery, our findings indicated that women who underwent cesarean section, particularly in the first stage of labor, scored significantly higher on the PPQ-II compared to those who experienced vaginal births. Cesarean sections, particularly emergency ones, are often perceived as traumatic due to their invasive nature and the potential for complications, both during and after the procedure (Carter et al., 2022; Ginter et al., 2022; Hüner et al., 2024). This is consistent with studies that have shown that women who experience emergency cesarean sections are at greater risk for developing post-traumatic stress symptoms compared to those who have vaginal deliveries (Carter et al., 2022; Suarez and Yakupova, 2023; Hüner et al., 2024). Interestingly, however, elective cesarean sections did not differ significantly from vaginal births in terms of PPQ-II scores, suggesting that the perception of trauma during childbirth may be more closely related to the circumstances of delivery rather than the method itself. Moreover, while complications during pregnancy and childbirth were considered as factors, such as hemorrhage, perineal trauma, and post-operative complications, their influence on PPQ-II scores was less pronounced. This could be due to the limited sample size of women experiencing these complications in our study. A larger and more diverse sample would be beneficial for exploring the impact of these factors in more depth. Nonetheless, the findings emphasize the complex nature of CB-PTSD development, with psychological outcomes being influenced by a multitude of factors, including physical, emotional, and situational aspects of the birth experience.

The analysis of the relationship between selected child health factors and maternal PTSD symptoms, as measured by the Polish PPQ-II, highlighted several findings that contribute to our understanding of how child-related factors may influence the development of PTSD symptoms in mothers. First, the comparison of PPQ-II scores between women who gave birth to premature infants versus those with full-term babies revealed that mothers of premature infants exhibited significantly higher scores across all PPQ-II domains, including Arousal, Avoidance & Intrusion, and total score. This finding is consistent with previous studies suggesting that premature birth can be a significant source of psychological distress for mothers, contributing to an increased risk of PTSD symptoms (Sanjuan et al., 2021; Laccetta et al., 2023). Premature birth is often associated with higher levels of medical uncertainty, prolonged hospital stays, and heightened emotional stress, which can serve as key risk factors for the development of post-traumatic stress symptoms in mothers (Gökçe et al., 2023).

Furthermore, the presence of a congenital defect in the child was also found to be associated with elevated PPQ-II scores, particularly in the Avoidance and Intrusion domains. Although the effect was moderate, this finding suggests that the psychological strain of having a child with a congenital defect can significantly impact maternal mental health. This is consistent with the literature, which has shown that mothers of children with congenital defects may experience higher levels of anxiety, stress, and trauma, as they may face an ongoing caregiving burden and concerns about their child’s health and future wellbeing (Roorda et al., 2022).

Another critical factor identified in the analysis was the hospitalization of the child in the intensive care unit (ICU). Mothers of children who required ICU care exhibited significantly higher PPQ-II scores across all domains, particularly in Avoidance and Intrusion. This result aligns with existing research that highlights the psychological impact of a child’s critical illness or hospitalization. The trauma of witnessing a child’s serious illness, especially in an intensive care setting, has been shown to significantly elevate the risk of PTSD symptoms in mothers (Bronner et al., 2008, 2010; Roorda et al., 2022). The emotional and physical toll of dealing with the medical complexities of a child’s ICU stay, combined with the feelings of helplessness and fear, may contribute to the heightened risk of developing PTSD-like symptoms (Bronner et al., 2010; Laccetta et al., 2023).

Additionally, the need for post-discharge medical care for the child was found to be a significant factor associated with higher PPQ-II scores. Women requiring long-term medical care for their child post-delivery exhibited the highest levels of PTSD symptoms, particularly in the areas of Avoidance and Intrusion. This is a notable finding, as the ongoing care demands and emotional stress related to caring for a child with chronic health issues can exacerbate psychological distress. Previous studies have suggested that the burden of long-term caregiving for children with health complications can result in persistent psychological strain, potentially leading to the development of PTSD symptoms in mothers (Khsim et al., 2022; Gökçe et al., 2023).

### 4.3 Strengths of the study

This study is one of the few to investigate the relationship between maternal factors, including sociodemographic characteristics, clinical variables, and child health factors, with P-PTSD symptoms in a large, diverse sample of women. The use of the Polish version of the PPQ-II offers a robust tool for measuring PTSD symptoms post-birth, adding valuable data to the body of knowledge on maternal mental health. By examining a wide range of factors, including the mode of delivery, complications, child health outcomes, and the quality of hospital amenities, this study provides a holistic view of the potential contributors to CB-PTSD symptoms. This allows for a more nuanced understanding of the different dimensions of maternal mental health following childbirth. The findings can inform clinical practice by identifying specific maternal and child health factors that are significantly associated with the risk of developing CB-PTSD symptoms. This can guide healthcare providers in offering targeted interventions to at-risk populations. The study contributes to the literature by presenting results specific to the Polish context, which can be valuable for understanding the local healthcare system’s impact on maternal mental health outcomes.

### 4.4 Limitations

While our study provides valuable insights into the factors influencing P-PTSD symptoms in postpartum women, several limitations should be considered. First, the study’s cross-sectional design restricts the ability to draw conclusions about causality. Longitudinal studies are necessary to determine the temporal relationships between maternal, child, and environmental factors and the onset of P-PTSD symptoms. Second, the sample size, though adequate for statistical analysis, was not large enough to capture all possible variations within specific subgroups, such as those with rare complications or more severe mental health issues. Sampling relied on voluntary public participation (convenience sampling) and was not based on a systematic selection process to ensure population representativeness. Additionally, the study relied on self-reported data, which may introduce biases such as social desirability or recall bias. Moreover, the cross-sectional design precludes causal inferences, limiting the ability to examine temporal relationships between risk factors and symptom development. Crucially, the assessment of CB-PTSD symptoms was based on self-report questionnaires (PPQ-II) rather than clinical diagnostic interviews. Therefore, the findings do not constitute a formal clinical diagnosis according to DSM-5 or ICD-11 criteria. Future research would benefit from incorporating structured clinical interviews to confirm diagnoses and enhance the clinical validity of the findings.

## 5 Conclusion

The Polish version of the PPQ-II proved to be a valid and reliable tool for assessing P-PTSD symptoms in postpartum women. It is important to emphasize that the norms established in this study are preliminary and based on a non-clinical sample. Their clinical applicability and generalizability require rigorous validation in larger, clinically confirmed, and geographically representative samples. The scale was sensitive to various maternal and child health factors, allowing for a nuanced understanding of the psychological consequences of childbirth. Given its comprehensive structure, the PPQ-II can be used effectively in clinical settings to screen for P-PTSD, enabling early identification of at-risk mothers and providing a basis for targeted interventions.

The study highlights those maternal factors such as economic status, mode of delivery, and the presence of complications that significantly influence the risk of developing P-PTSD symptoms. Child health factors, including the need for neonatal intensive care, also play a significant role in shaping maternal mental health outcomes. Based on the identified risk factors, healthcare providers should consider early screening for CB-PTSD among women with a history of complications, surgical deliveries, or children requiring intensive care. Tailored psychological interventions may help mitigate the psychological burden of childbirth and improve long-term mental health outcomes for mothers. The study underscores the need for systemic changes in maternal care, with particular attention to emotional support, quality of medical care, and the overall birth experience. This could contribute to reducing the incidence of P-PTSD among mothers, thereby improving maternal mental health outcomes at the population level.

## Data Availability

The raw data supporting the conclusions of this article will be made available by the authors, without undue reservation.
