# Correction: Validation of the Polish PPQ-II: sociodemographic and clinical correlates of perinatal PTSD

**DOI:** 10.3389/fpsyg.2026.1973915

**Published:** 2026-10-08

**Authors:** Anna Weronika Szablewska, Lucyna Wójcicka, Arkadiusz Prajzner, Agata Zdun-Ryżewska, Julia Burdecka, Dagmara Klasa-Mazurkiewicz

**Affiliations:** 1 Division of Obstetric and Gynaecological Nursing, Faculty of Health Sciences with the Institute of Maritime and Tropical Medicine, Medical University of Gdańsk, Gdańsk, Poland; 2 Institute of Psychology, Faculty of Pedagogy and Psychology, University of the National Education Commission, Krakow, Kraków City, Poland; 3 Division of Quality of Life Research, Department of Psychology, Faculty of Health Sciences with the Institute of Maritime and Tropical Medicine, Medical University of Gdańsk, Gdańsk, Poland; 4 Division of Gynecology and Obstetrics, Department of Gynecology, Obstetrics and Neonatology, Medical University of Gdańsk, Gdańsk, Poland

**Keywords:** women's mental health, perinatal post-traumatic stress disorder, psychometric properties, questionnaires, cross-cultural validation

The citation in the section [Section **2 Materials and methods**, *2.2 Data collection, 2.2.2 Postpartum PTSD Questionnaire II (PPQ-II)*] was erroneously written as [Goliczewska, A., and Szablewska, A. W. (2024). Analysis of biopsychosocial factors in relation to the procreation plans of Poles of reproductive age. *Nurs. Probl*. 32, 104–110. doi: 10.5114/ppiel.2024.140497].

It should be [Callahan, J. L., Borja, S. E., and Hynan, M. T. (2006). Modification of the Perinatal PTSD Questionnaire to enhance clinical utility. *J. Perinatol*. 26, 533–539. doi: 10.1038/sj.jp.7211562]

The text has been updated to read:

“Its structure initially refers to the DSM-IV criteria (American Psychiatric Association, 1994). Unlike the original version created by DeMier et al. (1996) in the revised version by Callahan et al. (2006) each item was rated on a five-point Likert scale ranging from 0 (“never”) to 4 (“frequently and for more than a month”). Higher total scores indicate greater PTSD symptom severity. Permission to use and validate the PPQ-II in Poland was obtained from the original authors (Callahan et al., 2006). The Polish version of the PPQ II questionnaire has been included in **Supplementary Appendix 1**.”

The citation in the section [Section **3 Results**, *3.1 Relevance*] was erroneously written as [Nardozza et al., 2025].

It should be [Field, A.P. (2018). *Discovering Statistics Using IBM SPSS Statistics*. 5th Edition, Sage: Newbury Park.]

The text has been updated to read:

“The high KMO test value (0.90) indicated that the sample (*N* = 273) was sufficiently large (Field, 2018).”

The citation in the section [Section **3 Results**, *3.1 Relevance*] was erroneously written as [Nardozza et al., 2025].

It should be [Field, A.P. (2018). *Discovering Statistics Using IBM SPSS Statistics*. 5th Edition, Sage: Newbury Park.]

The text has been updated to read:

“Cattell's factor extraction criterion (**Figure 1**) and Kaiser's criterion (**Table 1**) were equally met (Field, 2018).”

The citation in the section [Section **3 Results**, *3.2 Reliability*] was erroneously written as [Minister Zdrowia (2018). *Rozporzadzenie Ministra Zdrowia poz. 1756 z dnia 16 sierpnia 2018 r. w sprawie standardu organizacyjnego opieki okoloporodowej [Regulation of the Minister of Health, item 1756, of August 16, 2018, on the organizational standard of perinatal care]. Dz. Ustaw Rzeczyposp. Pol. 1–21 [Journal of Laws of the Republic of Poland, Nos. 1–21*]. Warsaw: Minister Zdrowia. Polish.].

It should be [Hernández-Martínez, A., Martínez-Vázquez, S., Rodríguez-Almagro, J., Khan, K. S., Delgado-Rodríguez, M., and Martínez-Galiano, J. M. (2021). Validation of perinatal post-traumatic stress disorder questionnaire for Spanish women during the postpartum period. *Sci. Rep*. 11:5567. doi: 10.1038/s41598-021-85144-2]

The text has been updated to read: “The exceptionally high reliability of the Polish version aligns with the similarly high values (αC = 0.90, ωM = 0.88, or αC = 0.90) reported for the Spanish (Hernández-Martínez et al., 2021), Portuguese (Alves et al., 2024), and English-language translations (Quinnell and Hynan, 1999).”

The citation in the section [Section **3 Results**, *3.2 Reliability*] was erroneously written as [Benzakour, L., Gayet-Ageron, A., Jubin, M., Suardi, F., Pallud, C., Lombard, F.-B., et al. (2022). Traumatic childbirth and birth-related post-traumatic stress disorder in the time of the COVID-19 pandemic: a prospective cohort study. *Int. J. Environ. Res. Public Health* 19:14246. doi: 10.3390/ijerph192114246.]

It should be [Alves, S., Claro, M., and Beato, A. (2024). Psychometric properties of the European portuguese version of the modified perinatal PTSD questionnaire (PPQ-II). *Curr*. *Psychol*. 43, 11865–11874. doi: 10.1007/s12144-023-05277-7]

The text has been updated to read: “The exceptionally high reliability of the Polish version aligns with the similarly high values (αC = 0.90, ωM = 0.88, or αC = 0.90) reported for the Spanish (Hernández-Martínez et al., 2021), Portuguese (Alves et al., 2024), and English-language translations (Quinnell and Hynan, 1999).”

The citation in the section [Section **3 Results**, *3.2 Reliability*] was erroneously written as [Ministerstwo Zdrowia (2024). *Przestrzeganie praw kobiet ciezarnych i rodzacych, funkcjonowanie standardu opieki okoloporodowej [Respect for the rights of pregnant and childbearing women, and the implementation of perinatal care standards*]. Warsaw: Odpowiedz MZ. Polish.].

It should be [Quinnell, F. A., and Hynan, M. T. (1999). Convergent and discriminant validity of the perinatal PTSD questionnaire (PPQ): a preliminary study. *J. Trauma. Stress* 12, 193–199. doi: 10.1023/A:1024714903950]

The text has been updated to read: “The exceptionally high reliability of the Polish version aligns with the similarly high values (αC = 0.90, ωM = 0.88, or αC = 0.90) reported for the Spanish (Hernández-Martínez et al., 2021), Portuguese (Alves et al., 2024), and English-language translations (Quinnell and Hynan, 1999).”

The citation in the section [Section **3 Results**, *3.3 Standardization*] was erroneously written as [Ozolins, U., Hale, S., Cheng, X., Hyatt, A., and Schofield, P. (2020). Translation and back-translation methodology in health research - a critique. *Expert Rev. Pharmacoecon*. *Outcomes Res*. 2069–2077. doi: 10.1080/14737167.2020.1734453].

It should be [Brzeziński J. (2023). *Metodologia badań psychologicznych: wydanie nowe*. Warsaw: Wydawnictwo Naukowe PWN.]

The text has been updated to read: “Despite the limited sample size, norms were developed for the Polish version of the PPQ-II. In light of the issue with the normal distribution of the total score [K-S(273) = 0.11, *p* < 0.001], the development of normative values was preceded by a non-linear transformation of the scores (Brzeziński, 2023).”

The citation in the section [Section **4 Discussion**, *4.1 The polish validation of the PPQ-II*] was erroneously written as [Heyne, C.-S., Kazmierczak, M., Souday, R., Horesh, D., Lambregtse-van, den Berg, M., et al. (2022). Prevalence and risk factors of birth-related posttraumatic stress among parents: a comparative systematic review and meta-analysis. *Clin. Psychol. Rev*. 94:102157. doi: 10.1016/j.cpr.2022.102157.]

It should be [Hernández-Martínez, A., Martínez-Vázquez, S., Rodríguez-Almagro, J., Khan, K. S., Delgado-Rodríguez, M., and Martínez-Galiano, J. M. (2021). Validation of perinatal post-traumatic stress disorder questionnaire for Spanish women during the postpartum period. *Sci. Rep*. 11:5567. doi: 10.1038/s41598-021-85144-2]

The text has been updated to read:

“To assess the psychometric properties of the Polish PPQ-II, we estimated its basic parameters, including reliability and validity. The Polish adaptation demonstrated excellent psychometric characteristics, with high internal consistency for the total score, as well as strong reliability for its subscales. These values are comparable to the previously validated Spanish (Hernández-Martínez et al., 2021), Portuguese (Alves et al., 2024) and English (Quinnell and Hynan, 1999) versions.”

The citation in the section [Section **4 Discussion**, *4.1 The polish validation of the PPQ-II*] was erroneously written as [Benzakour, L., Gayet-Ageron, A., Jubin, M., Suardi, F., Pallud, C., Lombard, F.-B., et al. (2022). Traumatic childbirth and birth-related post-traumatic stress disorder in the time of the COVID-19 pandemic: a prospective cohort study. *Int. J. Environ. Res. Public Health* 19:14246. doi: 10.3390/ijerph192114246.]

It should be [Alves, S., Claro, M., and Beato, A. (2024). Psychometric properties of the European portuguese version of the modified perinatal PTSD questionnaire (PPQ-II). *Curr. Psychol*. 43, 11865–11874. doi: 10.1007/s12144-023-05277-7]

The text has been updated to read:

“To assess the psychometric properties of the Polish PPQ-II, we estimated its basic parameters, including reliability and validity. The Polish adaptation demonstrated excellent psychometric characteristics, with high internal consistency for the total score, as well as strong reliability for its subscales. These values are comparable to the previously validated Spanish (Hernández-Martínez et al., 2021), Portuguese (Alves et al., 2024) and English (Quinnell and Hynan, 1999) versions.”

The citation in the section [Section **4 Discussion**, *4.1 The polish validation of the PPQ-II*] was erroneously written as [Horsch, A., Garthus-Niegel, S., Ayers, S., Chandra, P., Hartmann, K., Vaisbuch, E., et al. (2024). Childbirth-related posttraumatic stress disorder: definition, risk factors, pathophysiology, diagnosis, prevention, and treatment. *Am. J. Obstet. Gynecol*. 230, S1116–S1127. doi: 10.1016/j.ajog.2023.09.089.]

It should be [Quinnell, F. A., and Hynan, M. T. (1999). Convergent and discriminant validity of the perinatal PTSD questionnaire (PPQ): a preliminary study. *J. Trauma. Stress* 12, 193–199. doi: 10.1023/A:1024714903950]

The text has been updated to read: “To assess the psychometric properties of the Polish PPQ-II, we estimated its basic parameters, including reliability and validity. The Polish adaptation demonstrated excellent psychometric characteristics, with high internal consistency for the total score, as well as strong reliability for its subscales. These values are comparable to the previously validated Spanish (Hernández-Martínez et al., 2021), Portuguese (Alves et al., 2024) and English (Quinnell and Hynan, 1999) versions.”

The citation in the section [Section **4 Discussion**, *4.1 The polish validation of the PPQ-II*] was erroneously written as [Goliczewska, A., and Szablewska, A. W. (2024). Analysis of biopsychosocial factors in relation to the procreation plans of Poles of reproductive age. *Nurs. Probl*. 32, 104–110. doi: 10.5114/ppiel.2024.140497.]

It should be [Callahan, J. L., Borja, S. E., and Hynan, M. T. (2006). Modification of the Perinatal PTSD Questionnaire to enhance clinical utility. *J. Perinatol*. 26, 533–539. doi: 10.1038/sj.jp.7211562]

The text has been updated to read: “Finally, we examined the factorial structure of the questionnaire to verify its construct validity. The high Kaiser-Meyer-Olkin (KMO) test value (0.90) and the statistically significant Bartlett's test of sphericity confirmed the suitability of the dataset for exploratory factor analysis (EFA). Our findings indicate that, unlike the study by Callahan et al. (2006), the PPQ-II contains of two rather than three factors, which is consistent with results from the Italian (Nardozza et al., 2025), Portuguese (Alves et al., 2024), Turkish (Akik and Batigun, 2020), and French (Pierrehumbert et. al., 2004) versions of the scale.”

The citation in the section [Section **4 Discussion**, *4.1 The polish validation of the PPQ-II*] was erroneously written as [Benzakour, L., Gayet-Ageron, A., Jubin, M., Suardi, F., Pallud, C., Lombard, F.-B., et al. (2022). Traumatic childbirth and birth-related post-traumatic stress disorder in the time of the COVID-19 pandemic: a prospective cohort study. *Int. J. Environ. Res. Public Health* 19:14246. doi: 10.3390/ijerph192114246.]

It should be [Nardozza, O., Passaquindici, I., Persico, M. E., D'Andrea, A., Suttora, C., Fasolo, M., et al. (2025). The validation of the perinatal post-traumatic questionnaire in the italian population: risk and protective factors. *J. Clin. Med*. 14:704. doi: 10.3390/jcm14030704]

The text has been updated to read: “Finally, we examined the factorial structure of the questionnaire to verify its construct validity. The high Kaiser-Meyer-Olkin (KMO) test value (0.90) and the statistically significant Bartlett's test of sphericity confirmed the suitability of the dataset for exploratory factor analysis (EFA). Our findings indicate that, unlike the study by Callahan et al. (2006), the PPQ-II contains of two rather than three factors, which is consistent with results from the Italian (Nardozza et al., 2025), Portuguese (Alves et al., 2024), Turkish (Akik and Batigun, 2020), and French (Pierrehumbert et. al., 2004) versions of the scale.”

The citation in the section [Section **4 Discussion**, *4.1 The polish validation of the PPQ-II*] was erroneously written as [Bronner, M. B., Knoester, H., Bos, A. P., Last, B. F., and Grootenhuis, M. A. (2008). Follow-up after paediatric intensive care treatment: Parental posttraumatic stress. *Acta Paediatr*. 97, 181–186. doi: 10.1111/j.1651-2227.2007.00600.x.]

It should be [Alves, S., Claro, M., and Beato, A. (2024). Psychometric properties of the European Portuguese version of the modified perinatal PTSD questionnaire (PPQ-II). *Curr. Psychol*. 43, 11865–11874. doi: 10.1007/s12144-023-05277-7]

The text has been updated to read:

“Finally, we examined the factorial structure of the questionnaire to verify its construct validity. The high Kaiser-Meyer-Olkin (KMO) test value (0.90) and the statistically significant Bartlett's test of sphericity confirmed the suitability of the dataset for exploratory factor analysis (EFA). Our findings indicate that, unlike the study by Callahan et al., (2006), the PPQ-II contains of two rather than three factors, which is consistent with results from the Italian (Nardozza et al., 2025), Portuguese (Alves et al., 2024), Turkish (Akik and Batigun, 2020), and French (Pierrehumbert et. al., 2004) versions of the scale.”

The citation in the section [Section **4 Discussion**, *4.1 The polish validation of the PPQ-II*] was erroneously written as [Akik, B. K., and Batigun, A. D. (2020). Perinatal post traumatic stress disorder questionnaire-II (PPQ-II): adaptation, validity, and reliability study. *Dusunen Adam. J. Psychiatry Neurol. Sci*. 33, 340–350. doi: 10.14744/DAJPNS.2020.00102.]

It should be [Pierrehumbert, B., Borghini, A., Forcada-Guex, M., Jaunin, L., Muller-Nix, C. I., and Ansermet, F. (2004). Validation française d'un questionnaire de stress post-traumatique destiné aux parents d'enfants présentant un risque périnatal élevé. *Annales Medico-psychologiques - Ann Medico-Psychol*. 162, 711–721. doi: 10.1016/j.amp.2003.10.017]

The text has been updated to read:

“Finally, we examined the factorial structure of the questionnaire to verify its construct validity. The high Kaiser-Meyer-Olkin (KMO) test value (0.90) and the statistically significant Bartlett's test of sphericity confirmed the suitability of the dataset for exploratory factor analysis (EFA). Our findings indicate that, unlike the study by Callahan et al., (2006), the PPQ-II contains of two rather than three factors, which is consistent with results from the Italian (Nardozza et al., 2025), Portuguese (Alves et al., 2024), Turkish (Akik and Batigun, 2020), and French (Pierrehumbert et al., 2004) versions of the scale.”

The citation in the section [Section **4 Discussion**, *4.1 The polish validation of the PPQ-II*] was erroneously written as [Heyne, C.-S., Kazmierczak, M., Souday, R., Horesh, D., Lambregtse-van, den Berg, M., et al. (2022). Prevalence and risk factors of birth-related posttraumatic stress among parents: a comparative systematic review and meta-analysis. *Clin. Psychol. Rev*. 94:102157. doi: 10.1016/j.cpr.2022.102157.]

It should be [Hernández-Martínez, A., Martínez-Vázquez, S., Rodríguez-Almagro, J., Khan, K. S., Delgado-Rodríguez, M., and Martínez-Galiano, J. M. (2021). Validation of perinatal post-traumatic stress disorder questionnaire for Spanish women during the postpartum period. *Sci. Rep*. 11:5567. doi: 10.1038/s41598-021-85144-2]

The text has been updated to read: “Variability in the factor structure of the PPQ-II has been observed in previous research, both in terms of the number of factors and the distribution of items. While some validation studies support the original three-factor model (Hernández-Martínez et al., 2021), (Park et al., 2016) the assignment of items to specific factors differs across studies. Even in the original research, unexpected item loadings were reported (e.g., item 2, related to distressing memories, loaded onto the Avoidance factor (Callahan et al., 2006).”

The citation in the section [Section **4 Discussion**, *4.1 The polish validation of the PPQ-II*] was erroneously written as [American Psychiatric Association (n.d.). Available online at: https://psychiatryonline.org/doi/book/10.1176/appi.books. 9780890425787].

It should be [Park, Y. K., Ju, H. O., and Na, H. (2016). Reliability and validity of the Korean version of the perinatal post-traumatic stress disorder questionnaire. *jkan* 46, 29–38. doi: 10.4040/jkan.2016.46.1.29]

The text has been updated to read:

“Variability in the factor structure of the PPQ-II has been observed in previous research, both in terms of the number of factors and the distribution of items. While some validation studies support the original three-factor model (Hernández-Martínez et al., 2021), (Park et al., 2016) the assignment of items to specific factors differs across studies. Even in the original research, unexpected item loadings were reported (e.g., item 2, related to distressing memories, loaded onto the Avoidance factor (Callahan et al., 2006).”

The citation in the section [Section **4 Discussion**, *4.1 The polish validation of the PPQ-II*] was erroneously written as [Goliczewska, A., and Szablewska, A. W. (2024). Analysis of biopsychosocial factors in relation to the procreation plans of Poles of reproductive age. *Nurs. Probl*. 32, 104–110. doi: 10.5114/ppiel.2024.140497.]

It should be [Callahan, J. L., Borja, S. E., and Hynan, M. T. (2006). Modification of the Perinatal PTSD Questionnaire to enhance clinical utility. *J. Perinatol*. 26, 533–539. doi: 10.1038/sj.jp.7211562]

The text has been updated to read: “Variability in the factor structure of the PPQ-II has been observed in previous research, both in terms of the number of factors and the distribution of items. While some validation studies support the original three-factor model (Hernández-Martínez et al., 2021), (Park et al., 2016) the assignment of items to specific factors differs across studies. Even in the original research, unexpected item loadings were reported (e.g., item 2, related to distressing memories, loaded onto the Avoidance factor (Callahan et al., 2006).”

The original version of this article has been updated.

