# Supplementary material for: Validation of the Polish PPQ-II: sociodemographic and clinical correlates of perinatal PTSD
Source: Front Psychol. 2025 Oct 24;16:1698527. doi: 10.3389/fpsyg.2025.1698527 (PMC12592061; doi:10.3389/fpsyg.2025.1698527)
Supplement: Supplementary file 1 [file Table_1.pdf]

Appendix

S1 Appendix

Kwestionariusz okołoporodowego zespołu stresu pourazowego-II, ang. Perinatal Post Traumatic Stress Disorder Questionnaire-II (PPQ-II), J. L. Callahan, S. E. Borja, M. T. Hynan, 2006.

Tłumaczenie wersji polskiej: L. Wójcicka, A. Szablewska

Poniższe pytania dotyczą Twoich doświadczeń związanych z okresem ciąży, porodu i opieki nad noworodkiem. Jak często doświadczasz takich sytuacji?

Skala odpowiedzi i waga punktacji dla każdego pytania: (0) nigdy; (1); rzadko (zdarzyło się 1-2 razy); (2) czasami; (3) często, ale nie dłużej niż przez 1 miesiąc; (4) Bardzo często, dłużej niż przez miesiąc.

(1) Czy śniły Ci się koszmary o porodzie lub pobycie Twojego dziecka w szpitalu?

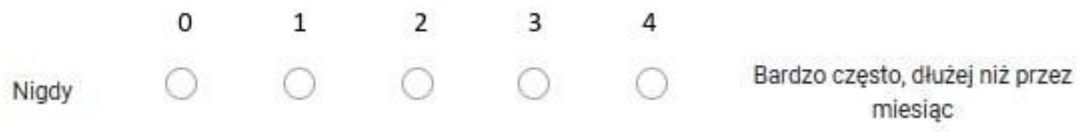

(2) Czy dręczyły Cię niepokojące wspomnienia o porodzie lub pobycie Twojego dziecka w szpitalu?

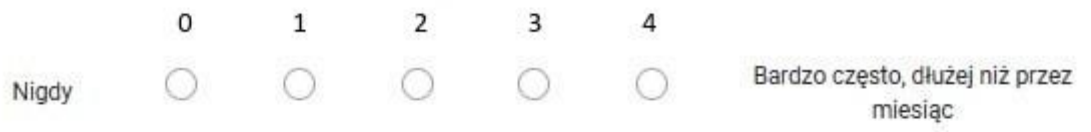

(3) Czy miewałaś nagłe nawracające wrażenie, jakby Twoje dziecko właśnie miało urodzić się ponownie?

	0	1	2	3	4	
Nigdy	○	○	○	○	○	Bardzo często, dłużej niż przez miesiąc

(4) Czy starałaś się unikać myślenia o porodzie lub pobycie twojego dziecka w szpitalu?

	0	1	2	3	4	
Nigdy	○	○	○	○	○	Bardzo często, dłużej niż przez miesiąc

(5) Czy unikałaś czynności, które mogłyby przywołać uczucia, które towarzyszyły Ci podczas porodu lub pobytu Twojego dziecka w szpitalu (np. unikanie oglądania programu telewizyjnego o dzieciach)?

	0	1	2	3	4	
Nigdy	○	○	○	○	○	Bardzo często, dłużej niż przez miesiąc

(6) Czy miałaś trudności z przypomnieniem sobie części pobytu Twojego dziecka w szpitalu?

	0	1	2	3	4	
Nigdy	○	○	○	○	○	Bardzo często, dłużej niż przez miesiąc

(7) Czy straciłaś zainteresowanie czynnościami, które zwykle wykonujesz (na przykład straciłaś zainteresowanie pracą lub rodziną)?

	0	1	2	3	4	
Nigdy	○	○	○	○	○	Bardzo często, dłużej niż przez miesiąc

(8) Czy czułaś się samotna i odizolowana od innych ludzi (na przykład czułaś, że nikt Cię nie rozumie)?

	0	1	2	3	4	
Nigdy	○	○	○	○	○	Bardzo często, dłużej niż przez miesiąc

(9) Czy trudniej było Ci odczuwać czułość lub miłość do innych osób?

	0	1	2	3	4	
Nigdy	○	○	○	○	○	Bardzo często, dłużej niż przez miesiąc

(10) Czy miałaś nietypowe trudności z zasypianiem lub utrzymaniem snu?

	0	1	2	3	4	
Nigdy	○	○	○	○	○	Bardzo często, dłużej niż przez miesiąc

(11) Czy byłaś bardziej niż zwykle drażliwa lub zła na innych?

	0	1	2	3	4	
Nigdy	○	○	○	○	○	Bardzo często, dłużej niż przez miesiąc

(12) Czy miałaś większe trudności z koncentracją niż przed porodem?

	0	1	2	3	4	
Nigdy	○	○	○	○	○	Bardzo często, dłużej niż przez miesiąc

(13) Czy czułaś się bardziej rozdrażniona (np. czy byłaś bardziej wrażliwa na hałas, lub łatwiej się przestraszałaś)?

	0	1	2	3	4	
Nigdy	○	○	○	○	○	Bardzo często, dłużej niż przez miesiąc

(14) Czy czułaś większe poczucie winy z powodu porodu, niż powinnaś?

	0	1	2	3	4	
Nigdy	○	○	○	○	○	Bardzo często, dłużej niż przez miesiąc

S2 Appendix. Factor loads, reliability and standardization of Polish PPQ-II measurement

Table S2.1. *Factor loads of the Polish PPQ-II measurement items*

Item	Original form<br>*Polish translation*	Arousal	Avoidance & Intrusion
1	Did you have bad dreams of giving birth or of your baby's hospital stay?<br>*Czy śniły Ci się koszmary o porodzie lub pobycie Twojego dziecka w szpitalu?*		.70
2	Did you have upsetting memories of giving birth or of your baby's hospital stay?<br>*Czy dręczyły Cię niepokojące wspomnienia o porodzie lub pobycie Twojego dziecka w szpitalu?*		.80
3	Did you have any sudden feelings as though your baby's birth was happening again?<br>*Czy miewałaś nagłe nawracające wrażenie, jakby Twoje dziecko właśnie miało urodzić się ponownie?*		.57
4	Did you try to avoid thinking about childbirth or your baby's hospital stay?<br>*Czy starałaś się unikać myślenia o porodzie lub pobycie twojego dziecka w szpitalu?*		.78
5	Did you avoid doing things that might bring up feelings you had about childbirth or your baby's hospital stay (e.g., not watching a TV show about babies)?<br>*Czy unikałaś czynności, które mogłyby przywołać uczucia, które towarzyszyły Ci podczas porodu lub pobytu Twojego dziecka w szpitalu (np. unikanie oglądania programu telewizyjnego o dzieciach)?*		.76
6	Were you unable to remember parts of your baby's hospital stay?<br>*Czy miałaś trudności z przypomnieniem sobie części pobytu Twojego dziecka w szpitalu?*	.78	
7	Did you lose interest in doing things you usually do (e.g., did you lose interest in your work or family)?<br>*Czy straciłaś zainteresowanie czynnościami, które zwykle wykonujesz (na przykład straciłaś zainteresowanie pracą lub rodziną)?*	.68	
8	Did you feel alone and removed from other people (e.g., did you feel like no one understood you)?<br>*Czy czułaś się samotna i odizolowana od innych ludzi (na przykład czułaś, że nikt Cię nie rozumie)?*	.80	
9	Did it become more difficult for you to feel tenderness or love with others?<br>*Czy trudniej było Ci odczuwać czułość lub miłość do innych osób?*	.78	
10	Did you have unusual difficulty falling asleep or staying asleep?<br>*Czy miałaś nietypowe trudności z zasypianiem lub utrzymaniem snu?*	.67	
11	Were you more irritable or angry with others than usual?<br>*Czy byłaś bardziej niż zwykle drażliwa lub zła na innych?*	.77	
12	Did you have greater difficulties concentrating than before you gave birth?<br>*Czy miałaś większe trudności z koncentracją niż przed porodem?*	.80	
13	Did you feel more jumpy (e.g., did you feel more sensitive to noise, or more easily startled)?<br>*Czy czułaś się bardziej rozdrażniona (np. czy byłaś bardziej wrażliwa na hałas, lub łatwiej się przestraszałaś)?*	.73	
14	Did you feel more guilty about the childbirth than you felt you should have felt?<br>*Czy czułaś większe poczucie winy z powodu porodu, niż powinnaś?*		.71

Note. Principal axis factoring with promax rotation was used.

Table S2.2. *Internal consistency of the Polish PPQ-II scales while removing items*

Item	Arousal		Avoidance & Intrusion		Total score	
	α_C	ω_M	α_C	ω_M	α_C	ω_M
1			.84	.86	.91	.91
2			.81	.83	.91	.91
3			.86	.87	.92	.91
4			.82	.84	.91	.91
5			.83	.85	.91	.91
6	.90	.90			.91	.91
7	.90	.90			.91	.91
8	.90	.89			.91	.91
9	.90	.89			.91	.91
10	.91	.90			.91	.91
11	.90	.89			.91	.91
12	.90	.90			.91	.91
13	.90	.90			.91	.91
14			.84	.86	.91	.91

α_C – *Alfa* Cronbacha, ω_M – *Omega* McDonalda

Table S2.3. *Standard values of the Polish PPQ-II total score*

Raw score [*Min–Max*]	Standard ten scale
0	1
1–3	2
4–6	3
7–10	4
11–16	5
17–25	6
26–32	7
33–41	8
42–49	9
50–56	10

S3 Appendix. Analysis of Polish PPQ-II results broken down into sociodemographic variables

Table S3.1. *Difference analysis of Polish PPQ-II results broken down into education, economic status and relationship variables*

PPQ-II	Education						z	p	r_g
	Non-higher			Higher					
	N	*M*	*SD*	*N*	*M*	*SD*			
Arousal	56	13.63	8.58	217	13.39	8.67	.20	.841	.02
Avoidance & Intrusion	56	5.71	5.45	217	5.74	6.02	.19	.846	.02
Total score	56	19.34	12.49	217	19.13	13.15	.31	.756	.03
PPQ-II	Economic status						z	p	r_g
	Non-well			Well					
	N	*M*	*SD*	*N*	*M*	*SD*			
Arousal	48	16.73	8.83	225	12.74	8.45	**2.88**	**.004**	**.26**
Avoidance & Intrusion	48	6.88	5.96	225	5.49	5.87	1.71	.087	.16
Total score	48	23.60	13.10	225	18.23	12.81	**2.72**	**.007**	**.25**
PPQ-II	Relationship						z	p	r_g
	Non-formal			Formal					
	N	*M*	*SD*	*N*	*M*	*SD*			
Arousal	56	14.48	8.68	213	13.12	8.66	1.13	.260	.10
Avoidance & Intrusion	56	5.13	5.31	213	5.85	6.01	.61	.543	.05
Total score	56	19.61	12.87	213	18.98	13.04	.43	.668	.04
PPQ-II	Mother age						z	p	r_g
	Less than 35 y.a.			35 and more y.a.					
	N	*M*	*SD*	*N*	*M*	*SD*			
Arousal	226	13.33	8.61	47	13.98	8.86	.49	.626	.05
Avoidance & Intrusion	226	5.93	5.81	47	4.79	6.30	1.79	.073	.17
Total score	226	19.26	12.90	47	18.77	13.58	.34	.735	.03

N – number of observations, *M* – mean, *SD* – standard deviation, z – Mann-Whitney test result, p – significance, r_g – effect size

Table S3.2. *Difference analysis of Polish PPQ-II results broken down into place of residence variable*

PPQ-II	Place of residence									$H_{(2)}$	p	ε^2
	Rural			Small city up to 500k inhabitants			Big city over 500k inhabitants					
	N	*M*	*SD*	*N*	*M*	*SD*	*N*	*M*	*SD*			
Arousal	34	14.94	9.40	99	14.04	9.07	140	12.65	8.10	1.67	.415	.01
Avoidance & Intrusion	34	6.85	6.72	99	5.72	5.93	140	5.47	5.68	1.25	.534	.00
Total score	34	21.79	14.92	99	19.76	13.57	140	18.12	12.04	1.46	.482	.01

Note. Post-hoc analysis wasn't performed due to non-significant H tests results.
N – number of observations, *M* – mean, *SD* – standard deviation, *H* – Kruskal-Wallis test result, p – significance, ε^2 – effect size

Table S3.3 *Correlation analysis of Polish PPQ-II results with number of pregnancies and number of given births variables*

PPQ-II	Pregnancies		Given births	
	r_s	p	r_s	p
Arousal	.02	.702	-.05	.368
Avoidance & Intrusion	-.01	.875	**-.12**	**.045**
Total score	.02	.797	-.08	.215

r_s – Spearman's coefficient, p – significance

S4 Appendix. Analysis of Polish PPQ-II results broken down into mother and child related variables

Table S4.1 *Difference analysis of Polish PPQ-II results broken down mother related variables*

PPQ-II	Miscarriage						*z*	*p*	r_g
	Absence			Occurrence					
	N	*M*	*SD*	*N*	*M*	*SD*			
Arousal	230	13.07	8.40	43	15.44	9.69	1.42	.155	.14
Avoidance & Intrusion	230	5.30	5.46	43	8.07	7.51	**2.20**	**.028**	**.21**
Total score	230	18.36	12.24	43	23.51	15.96	1.74	.083	.17
PPQ-II	Intrapartum complications Kristeller's maneuver						*z*	*p*	r_g
	Absence			Occurrence					
	N	*M*	*SD*	*N*	*M*	*SD*			
Arousal	253	13.34	8.56	20	14.65	9.70	.41	.683	.06
Avoidance & Intrusion	253	5.55	5.71	20	8.00	7.80	1.29	.198	.17
Total score	253	18.90	12.66	20	22.65	16.68	.70	.482	.09
PPQ-II	Intrapartum complications Shoulder release maneuver						*z*	*p*	r_g
	Absence			Occurrence					
	N	*M*	*SD*	*N*	*M*	*SD*			
Arousal	268	13.40	8.61	5	15.40	11.19	.33	.740	.09
Avoidance & Intrusion	268	5.65	5.85	5	10.00	7.97	1.42	.155	.37
Total score	268	19.06	12.91	5	25.40	17.56	.89	.374	.23
PPQ-II	Near-term complications Postpartum hemorrhage						*z*	*p*	r_g
	Absence			Occurrence					
	N	*M*	*SD*	*N*	*M*	*SD*			
Arousal	252	13.21	8.44	21	16.14	10.57	1.08	.279	.14
Avoidance & Intrusion	252	5.60	5.81	21	7.38	6.84	1.23	.220	.16
Total score	252	18.81	12.67	21	23.52	16.16	1.12	.263	.15
PPQ-II	Further-term complications Extensive perineal trauma						*z*	*p*	r_g
	Absence			Occurrence					
	N	*M*	*SD*	*N*	*M*	*SD*			
Arousal	266	13.32	8.59	7	17.86	10.14	1.19	.234	.26
Avoidance & Intrusion	266	5.66	5.84	7	8.57	7.89	1.09	.276	.24
Total score	266	18.98	12.85	7	26.43	17.22	1.17	.241	.26
PPQ-II	Further-term complication of perineal wound healing						*z*	*p*	r_g
	Absence			Occurrence					
	N	*M*	*SD*	*N*	*M*	*SD*			
Arousal	271	13.46	8.66	2	10.50	2.12	0.25	.805	.10
Avoidance & Intrusion	271	5.75	5.92	2	4.00	1.41	0.07	.946	.03
Total score	271	19.21	13.04	2	14.50	0.71	0.31	.753	.13
PPQ-II	Further-term cesarean section complication						*z*	*p*	r_g
	Absence			Occurrence					
	N	*M*	*SD*	*N*	*M*	*SD*			
Arousal	270	13.44	8.67	3	13.67	6.51	.19	.851	.06
Avoidance & Intrusion	270	5.72	5.90	3	6.67	7.37	.38	.708	.13
Total score	270	19.16	13.02	3	20.33	13.65	.27	.788	.09
PPQ-II	Prolonged hospital stay						*z*	*p*	r_g
	Absence			Occurrence					
	N	*M*	*SD*	*N*	*M*	*SD*			
Arousal	207	13.44	8.56	66	13.44	8.93	.02	.983	.00
Avoidance & Intrusion	207	5.18	5.80	66	7.47	5.93	**3.22**	**.001**	**.26**
Total score	207	18.62	12.87	66	20.91	13.35	1.23	.218	.10

N – number of observations, *M* – mean, *SD* – standard deviation, *z* – Mann-Whitney test result, *p* – significance, r_g – effect size

Table S4.2

Difference analysis of Polish PPQ-II results broken down into mode of birth, near-term complications (curettage) and facilities during delivery

PPQ-II	Mode of birth						z	p	r_g
	Vaginal birth			Caesarean section					
	N	*M*	*SD*	*N*	*M*	*SD*			
Arousal	137	12.19	8.83	136	14.70	8.28	**2.59**	**.010**	**.18**
Avoidance & Intrusion	137	4.85	5.64	136	6.62	6.05	**2.90**	**.004**	**.20**
Total score	137	17.04	12.72	136	21.32	12.96	**2.94**	**.003**	**.21**
PPQ-II	Near-term complications (Curettage)						z	p	r_g
	Absence			Occurrence					
	N	*M*	*SD*	*N*	*M*	*SD*			
Arousal	249	12.98	8.51	24	18.17	8.73	**2.76**	**.006**	**.34**
Avoidance & Intrusion	249	5.47	5.75	24	8.50	6.81	**2.09**	**.036**	**.26**
Total score	249	18.45	12.72	24	26.67	13.70	**2.82**	**.005**	**.35**
PPQ-II	Facilities during delivery						z	p	r_g
	Absence			Occurrence					
	N	*M*	*SD*	*N*	*M*	*SD*			
Arousal	45	17.40	8.80	228	12.66	8.41	**3.33**	**.001**	**.31**
Avoidance & Intrusion	45	9.11	6.18	228	5.07	5.62	**4.23**	**<.001**	**.40**
Total score	45	26.51	13.43	228	17.72	12.44	**4.02**	**<.001**	**.38**

N – number of observations, *M* – mean, *SD* – standard deviation, *z* – Mann-Whitney test result, *p* – significance, r_g – effect size

Table S4.3

Difference analysis of Polish PPQ-II results broken down into mode of birth details

PPQ-II	Detail mode of birth												$H_{(3)}$	p	ε^2	post hoc
	Vaginal birth (1)			Caesarean section 1 stage (2)			Caesarean section 2 stage (3)			Caesarean section planned (4)						
	N	*M*	*SD*	*N*	*M*	*SD*	*N*	*M*	*SD*	*N*	*M*	*SD*				
Arousal	137	12.19	8.83	55	16.11	8.79	25	14.52	8.18	46	13.22	8.01	**9.34**	**0.25**	**.04**	**1<2**
Avoidance & Intrusion	137	4.85	5.64	55	7.84	6.65	25	8.08	6.11	46	4.50	4.92	**18.01**	**<.001**	**.07**	**1,4<2,3**
Total score	137	17.04	12.72	55	23.95	13.90	25	22.60	13.74	46	17.72	11.31	**13.09**	**.004**	**.05**	**1<2**

Note. Post hoc analyses were performed with multiple comparison adjusting Bonferroni's correction.

N – number of observations, *M* – mean, *SD* – standard deviation, *H* – Kruskal-Wallis test result, *p* – significance, ε^2 – effect size

Table S4.4

Difference analysis of Polish PPQ-II results broken down into duration of last pregnancy, child congenital anomaly and child ICU hospitalization

PPQ-II	Duration of last pregnancy						z	p	r_g
	Premature			On-time					
	N	*M*	*SD*	*N*	*M*	*SD*			
Arousal	54	16.20	9.20	219	12.76	8.38	**2.50**	**.012**	**.22**
Avoidance & Intrusion	54	9.31	6.68	219	4.85	5.35	**4.40**	**<.001**	**.39**
Total score	54	25.52	14.55	219	17.61	12.12	**3.56**	**<.001**	**.31**
**PPQ-II**	**Child congenital defect**						z	p	r_g
	Absence			Occurrence					
	N	*M*	*SD*	*N*	*M*	*SD*			
Arousal	265	13.28	8.63	8	18.75	7.76	1.82	.069	.38
Avoidance & Intrusion	265	5.61	5.87	8	9.88	5.72	**2.18**	**.029**	**.45**
Total score	265	18.89	12.94	8	28.63	11.80	**2.15**	**.031**	**.45**
**PPQ-II**	**Child ICU hospitalization**						z	p	r_g
	Absence			Occurrence					
	N	*M*	*SD*	*N*	*M*	*SD*			
Arousal	233	12.82	8.45	40	17.03	8.95	**2.89**	**.004**	**.29**
Avoidance & Intrusion	233	4.72	5.33	40	11.65	5.66	**6.48**	**<.001**	**.64**
Total score	233	17.54	12.26	40	28.68	13.23	**4.78**	**<.001**	**.47**

N – number of observations, *M* – mean, *SD* – standard deviation, *z* – Mann-Whitney test result, *p* – significance, r_g – effect size

Table S4.5

Difference analysis of Polish PPQ-II results broken down into additional post-discharge medical care

PPQ-II	Additional post-discharge medical care									$H_{(2)}$	p	ε^2	post hoc
	Absence (1)			Short-term (2)			Long-term (3)						
	N	*M*	*SD*	*N*	*M*	*SD*	*N*	*M*	*SD*				
Arousal	174	12.53	8.08	53	13.49	9.53	46	16.80	8.96	**8.90**	**.012**	**.03**	**1<3**
Avoidance & Intrusion	174	4.30	4.81	53	5.58	5.85	46	11.33	6.47	**41.45**	**<.001**	**.15**	**1,2<3**
Total score	174	16.83	11.39	53	19.08	13.74	46	28.13	14.19	**22.98**	**<.001**	**.08**	**1,2<3**

Note. Post hoc analyses were performed with multiple comparison adjusting Bonferroni's correction.

N – number of observations, *M* – mean, *SD* – standard deviation, *H* – Kruskal-Wallis test result, *p* – significance, ε^2 – effect size
